# Relationship Between Residency Match Distance From Medical School and Virtual Application, School Characteristics, and Specialty Competitiveness

**DOI:** 10.7759/cureus.38782

**Published:** 2023-05-09

**Authors:** Ammar Hasnie, Usman Hasnie, Benjamin Nelson, Isabella Aldana, Carlos Estrada, Winter Williams

**Affiliations:** 1 Department of Medicine, University of Alabama at Birmingham Heersink School of Medicine, Birmingham, USA; 2 Medicine Service, Birmingham Veterans Affairs Medical Center, Birmingham, USA

**Keywords:** graduate medical education, professional practice location, residency application, covid-19, internship and residency, geography

## Abstract

Introduction: The COVID-19 pandemic has disrupted the residency match process by eliminating away rotations and changing from in-person to virtual interviews. In this study, we explore the impact of the COVID-19 pandemic on the geographic match distance of United States (US) senior medical students across all specialties.

Methods: We collected publicly available student match data between 2018 and 2021 from US allopathic medical schools and calculated match distance between medical school and residency training using a novel metric - the “match space.” Match space was codified by whether the student matched at their home institution, home state, adjacent state, same or adjacent US census division (non-adjacent state) or skipped at least one US census division. Adjusting for covariates, ordinal logistic regression correlated school and specialty characteristics with match distance pre- and post-pandemic for all specialties. We defined and ranked specialty competitiveness using predictive values from factor analysis.

Results: A total of 34,672 students representing 66 medical schools from 28 states matched into 26 specialties in 50 states and Canada. Fifty-nine percent of students were from public institutions, and 27% of schools ranked in the top 40 for research. The mean percentage of in-state students by school was 60.3% (range 3-100%). Match space was lower after the pandemic (adjusted odds ratio (OR) 0.94, 95% CI 0.90-0.98; p=0.006), from schools with higher in-state percentages (OR 0.74, 95% CI 0.72-0.76), from top National Institutes of Health-funded institutions (OR 0.88, 95% CI 0.85-0.92), from the Northeast (OR 0.71, 95% CI 0.67-0.75; Midwest reference), and the West (OR 0.67, 95% 0.60-0.74). Match space was higher for students graduating from private schools (OR 1.11, 95% CI 1.05-1.19), from the South (OR 1.62, 95% CI 1.2-1.33), and matching into more competitive specialties (OR 1.08, 95% CI 1.02-1.14). The top five most competitive specialties were Plastic Surgery, Neurosurgery, Dermatology, Orthopedic Surgery, and Otolaryngology. Internal Medicine ranked eighth.

Conclusions: After the COVID-19 pandemic, students graduating from US allopathic schools matched closer to their home institution. Students attending public schools, schools with more in-state matriculants, and schools with higher research rankings also matched closer to their home institutions. Specialty competitiveness and US census region also impacted match distance. Our study adds insight into how geographic match patterns were influenced by school, specialty choice, and the pandemic.

## Introduction

In the United States (US), senior medical students and residency programs utilize the National Resident Matching Program (NRMP) to interview and fill residency positions each year [1]. The COVID-19 pandemic has disrupted this process by eliminating away rotations and changing from in-person to virtual interviews [2]. Despite efforts to optimize the virtual experience, it remains challenging for programs to gauge genuine applicant interest and for applicants to obtain a sense of a program’s culture and environment [3-5]. The transition to a virtual residency match has yielded significant benefits for applicants through reduced travel costs and more schedule flexibility, yet the reduced cost and general process uncertainty have led applicants to apply and interview with more programs, resulting in record high-rank order list lengths for programs and applicants alike [6,7].

These changes have exacerbated the pre-existing trend of application inflation, which led the Electronic Residency Application Service (ERAS®) to introduce a supplemental application for a growing number of specialties since the 2022 match cycle. In addition to their most meaningful experiences, applicants provide geographic preferences and “preference signals” to identify a limited number of preferred programs [8]. These changes reflect the growing recognition of the importance of geography as a factor in the residency match process [9,10]. We have previously shown that school and specialty characteristics impact geography; however, little is known about how adaptations to the COVID-19 pandemic have influenced residency match geography across all specialties [11]. As we conducted this study, Cotner et. al. found supporting evidence that the virtual match process would result in students staying closer to their home institutions, but their analysis was limited to the same state versus different states [12]. We and others have found varying results for different specialties: no difference in matched distance for plastic surgery [13], neurology [14], or cardiology fellowship [15]; for otolaryngology [16], dermatology [17], and orthopedic surgery [18], students matched closer to their home institution.

Our study aimed to investigate pre- and post-pandemic trends in the geographic placement of senior allopathic medical students for post-graduate training and to determine which factors impacted where applicants matched across all specialties. To do so, we utilized a metric that we called “match space” to quantify the non-linear geographic relationship between medical schools and residency programs [11].

Abstracts of earlier versions of this study were presented at Association of American Medical Colleges (November 8-10, 2021, virtual meeting), the Southern Society of General Internal Medicine Regional Meeting (February 9-10, 2022, virtual meeting), the Society of General Internal Medicine, Orlando, FL (April 6-9, 2022), and the Alliance for Academic Internal Medicine (AAIM), Academic Internal Medicine Week 2022, Charlotte, NC (April 11, 2022).

## Materials and methods

Study design, setting, and participants

**Figure 1:**
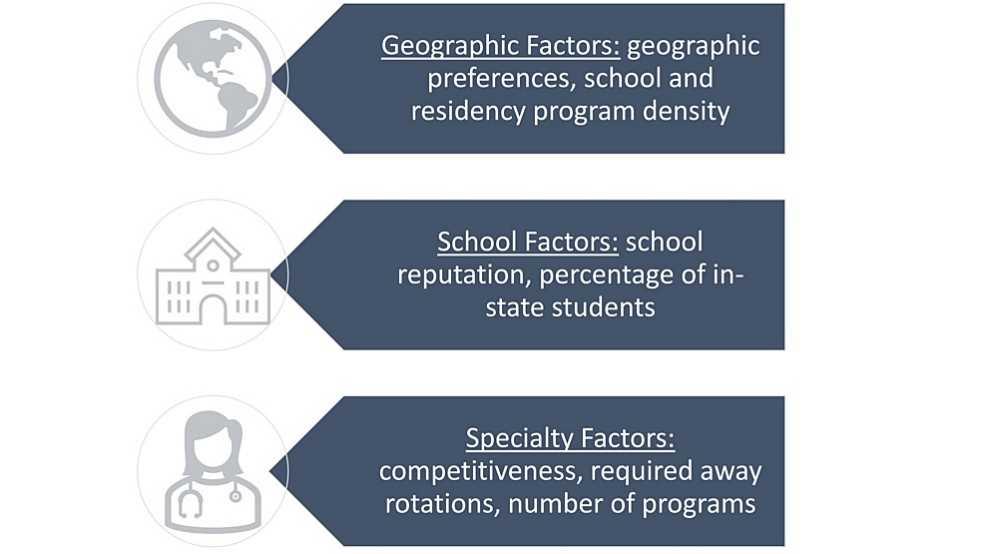
Conceptual Framework of Structural Factors Affecting Residency Match Placement.

We include the post-COVID-19 matched data to our prior publication with pre-COVID-19 matched data [11]; see the conceptual framework in Figure 1.

We included students graduating from US allopathic medical schools who were matched into residency programs during in-person interviews (pre-COVID-19, 2018-2020) and during virtual interviews (post-COVID-19, 2021) for all specialties. We excluded medical schools without publicly available match lists or schools with fewer than 50 graduating students. We also excluded students who matched into combined residencies, interventional radiology, oral and maxillofacial surgery, child psychiatry, non-residency fellowships, or preliminary-only positions. We collected matched residency program data for each student.

We obtained medical school characteristics from publicly available datasets. Public/private medical school designation was obtained from the Medical School Admission Requirements™ (MSAR®) online database [19]. School reputation was determined using a surrogate of the top 40 National Institutes of Health (NIH) funding based on US News and World Report rankings [20]. Finally, the percentage of in-state matriculants was obtained from the Association of American Medical Colleges [21].

Specialty characteristics including US senior applicants per position, US senior fill rate, and number of post-graduate year (PGY) positions available were collected using 2021 NRMP data [7]. The salary was collected from the 2020 annual physician compensation reports [22,23].

Statistical analysis

To examine specialty competitiveness, we used exploratory factor analysis of the specialty characteristics, retained items with Eigenvalues ≥ 1.0, and rotated factor loading > 0.4. Then, we ranked specialties based on the predictive value of the factor with the greatest Eigenvalue. We obtained Spearman rho correlations of the characteristics and assessed internal consistency reliability with Cronbach’s alpha (and considered alpha > 0.9 to be excellent and > 0.8 to be good).

We use the same statistical approach as in our prior study [11]. Match space was codified by whether the student matched at their home institution, home state, adjacent state, same or adjacent US census division (non-adjacent state), or skipped at least one US census division (5-point ordinal scale). We used ordinal logistic regression to explore the relationship between the match space and the following variables: pre-and post-COVID-19 match, school type, top 40 NIH ranking, percentage of in-state matriculants, US census region, and specialty competitiveness. Instead of using the full range for continuous variables (e.g. percentage of in-state matriculants) and for a more parsimonious interpretation of the statistical analyses, we collapsed data into quartiles [11]. A positive odds ratio meant that the match space was incrementally larger on the 5-point scale. All analyses were performed using StataCorp (2021, Stata Statistical Software: Release 17, College Station, TX: StataCorp LLC) with the logit module; we used a significance level of P<0.05.

## Results

We included 35,065 students (26,102 pre-COVID-19 for the match year 2018-2020; 8,963 post-COVID-19 for the match year 2021) from 66 medical schools, representing 28 states across the four US census regions and nine divisions; Table 1 shows studied variables.

**Table 1:** Characteristics of Included Graduates from 66 US Medical Schools Matching into US Residency Programs (n=35,065), Pre- (2018-2020) and Post-COVID-19 Pandemic (2021). Percentages may not add up to 100% due to missing data. NIH = National Institute of Health, PGY1 = post-graduate year. Procedural: Anesthesiology, Cardiothoracic Surgery, Emergency Medicine, General Surgery, Neurosurgery, Obstetrics/Gynecology, Ophthalmology, Orthopedic Surgery, Otolaryngology, Plastic Surgery, Urology, Vascular Surgery. Non-procedural: Child Neurology, Dermatology, Diagnostic Radiology, Family Medicine, Internal Medicine, Neurology, Pathology, Pediatrics, Physical Medicine and Rehabilitation, Psychiatry, Radiation Oncology. Competitiveness is defined by Factor Analysis.

Characteristic	Value
Year, n (%)	
2018	8,649 (24.67)
2019	8,726 (24.89)
2020	8,727 (24.89)
2021	8,963 (25.56)
School	
Type, n (%)	
Private	14,189 (40.46)
Public	20,876 (59.54)
Top 40 NIH research funding, n (%)	
Yes	10,866 (30.99)
No	24,199 (69.01)
% of In State Students Quartile, mean (SD)	
Quartile 1	19.21 (10.07)
Quartile 2	48.87 (7.38)
Quartile 3	76.59 (6.73)
Quartile 4	90.97 (5.47)
Overall	58.38 (28.68)
US Census Region, n (%)	
Northeast (Divisions 1, 2)	10,326 (29.45)
Midwest (Divisions 3, 4)	10,681 (30.46)
South (Divisions 5-7)	12,570 (35.85)
West (Divisions 8, 9)	1,488 (4.24)
US Census Division, n (%)	
Division 1	1,934 (5.52)
Division 2	8,392 (23.93)
Division 3	7,140 (20.36)
Division 4	3,541 (10.10)
Division 5	5,828 (16.62)
Division 6	2,239 (6.39)
Division 7	4,503 (12.84)
Division 8	230 (0.66)
Division 9	1,258 (3.59)
Specialty	
Procedural, n (%)	
Yes	14,256 (40.66)
No	20,809 (59.34)
Competitiveness, Factor Analysis, n (%)	
Low	10,843 (30.92)
Medium	9,230 (26.32)
High	10,563 (30.12)
Very High	3,370 (9.61)
US Senior Applicants per Position, mean (SD)	
Quartile 1	0.45 (0.04)
Quartile 2	0.69 (0.02)
Quartile 3	0.83 (0.08)
Quartile 4	1.06 (0.13)
Overall	0.73 (0.23)
% US Senior Fill Rate, mean (SD)	
Quartile 1	37.80 (3.33)
Quartile 2	58.29 (5.03)
Quartile 3	66.35 (1.63)
Quartile 4	81.53 (6.93)
Overall	58.87 (16.85)
Number of PGY1 Positions, mean (SD)	
Quartile 1	7,419 (1,876)
Quartile 2	2,558 (394)
Quartile 3	1,369 (185)
Quartile 4	501 (233)
Overall	3,337 (2,993)
Salary, mean (SD)	
Quartile 1	$ 266,473 (15,355)
Quartile 2	$ 309,503 (15,962)
Quartile 3	$ 382,171 (39,000)
Quartile 4	$ 507,784 (79,338)
Overall	$ 357,971 (105,134)
Matched Space, n (%)	
Home institution	5,700 (16.26)
Home state	6,631 (18.91)
Adjacent state	5,114 (14.58)
Same or continuous US Census Division and not adjacent state	8,844 (25.22)
Skips >=1 US Census Division	8,776 (25.03)
Total	35,065 (100)

Table 2 shows matched specialties.

**Table 2:** Characteristics of Included Graduates from 66 US Medical Schools Matching into US Residency Programs by Specialty (n=35,065), 2018-2021.

Specialty	Number (%)
Anesthesiology	2,284 (6.51)
Cardiothoracic Surgery	70 (0.20)
Child Neurology	215 (0.61)
Dermatology	729 (2.08)
Diagnostic Radiology	1,407 (4.01)
Emergency Medicine	3,277 (9.35)
Otolaryngology	538 (1.53)
Family Medicine	3,384 (9.65)
General Surgery	2,246 (6.41)
Internal Medicine	7,323 (20.88)
Neurosurgery	388 (1.11)
Neurology	975 (2.78)
Obstetrics/Gynecology	2,159 (6.16)
Ophthalmology	844 (2.41)
Orthopedic Surgery	1,426 (4.07)
Plastic Surgery	289 (0.82)
Pathology	418 (1.19)
Pediatrics	3,574 (10.19)
Physical Medicine and Rehabilitation	435 (1.24)
Psychiatry	2,057 (5.87)
Radiation Oncology	292 (0.83)
Urology	632 (1.80)
Vascular Surgery	103 (0.29)
Total	35,065 (100)

Factor analysis, specialty competitiveness

Among the 21 specialties, 10 of 14 variables had correlation coefficients of 0.8-0.9 (all p<0.001). Factor 1 explained 58% of the variance (Eigenvalue 8.2) and comprises a percentage of US senior fill rate, Step 1 score, Step 2 score, percentage of Alpha Omega Alpha Honor Society (AOA), scholarship, the percentage from top 40 NIH funding school, and salary potential (data not shown). Factor 2 explained 17% of the variance (Eigenvalue 2.3) and comprises the number of PGY-1 positions available, number of programs, total number of applicants, and total number of US applicants (data not shown). Factor 3 explained 10% of the variance (Eigenvalue 1.5) and comprises US senior applicants per position and applicants/position. The internal consistencies were excellent (Factor 1, Cronbach’s alpha = 0.94; Factor 2, Cronbach’s alpha = 0.97). Child Neurology and Ophthalmology had incomplete data and were not included in the analysis.

Based on the ranking of the predictive value for Factor 1, the top five most competitive specialties were Plastic Surgery, Neurosurgery, Dermatology, Orthopedic Surgery, and Otolaryngology. Internal Medicine ranked eighth. The least competitive specialties were Pediatrics, Neurology, Psychiatry, Pathology, Family Medicine, and Physical Medicine and Rehabilitation, see Table 3.

**Table 3:** Specialty Rank Competitiveness Based on Predicted Value of Factor 1 from Factor Analysis. AOA = Alpha Omega Alpha Honor Society, NIH = National Institute of Health. The scholarship is defined as the mean number of abstracts, presentations, and publications. Child Neurology and Ophthalmology had incomplete data (not included in the ranking).

Specialty	Competitiveness Predicted Value Factor 1	% US Senior Fill Rate	Salary	Step 1	Step 2	% AOA	% Top 40 NIH	Scholarship
Plastic Surgery	Very High	91.7	$539,208	249	256	43	34.2	19.1
Neurosurgery	Very High	87.5	$746,544	248	252	39	39	23.4
Dermatology	Very High	74.2	$449,494	248	256	47.4	41.3	19
Orthopedic Surgery	Very High	80.8	$605,330	248	255	40.3	33.6	14.3
Otolaryngology	Very High	88.6	$472,273	248	256	38.1	40.8	13.7
Urology	High	95	$472,941	243	247	31.4	32.3	10.1
Radiation Oncology	High	65.1	$516,016	243	250	22.3	46.4	18.3
Internal Medicine	High	40.2	$280,088	235	248	17.4	33.6	6.2
Cardiothoracic surgery	High	84.2	$668,350	243	248	18.5	48.1	7.6
General Surgery	High	67.3	$439,824	237	249	18.5	29.9	7.1
Diagnostic Radiology	Medium	66.7	$485,460	241	249	18.3	26	6.4
Vascular Surgery	Medium	81.3	$534,508	239	247	13.1	39.3	10.5
Obstetrics/Gynecology	Medium	75.5	$353,063	232	248	16.6	29.7	6
Emergency Medicine	Medium	64.3	$354,615	233	247	11.8	27.7	4.3
Anesthesiology	Medium	68.3	$445,533	234	246	9.4	30.6	5.2
Pediatrics	Low	60.4	$243,253	228	245	12.3	26.9	4.9
Neurology	Low	46.5	$322,085	232	245	14.3	35.2	7.2
Psychiatry	Low	61.2	$297,166	227	241	6.8	29.6	5.6
Pathology	Low	33.8	$340,873	233	242	11	36.3	7.3
Family Medicine	Low	33.1	$261,536	221	238	6.4	26.7	3.3
Physical Medicine and Rehabilitation	Low	53.6	$354,457	228	241	5	21.7	5.5

Matched space

Overall, 5,700 (16.26%) students matched at their home institution, 6,631 (18.91%) matched within the same state as their medical school, 5,114 (14.58%) matched into adjacent states, 8,844 (25.22%) matched in the same or contiguous division (non-adjacent state), and 8,776 (25.03%) matched skipping at least one division, see Table 1.

**Figure 2:**
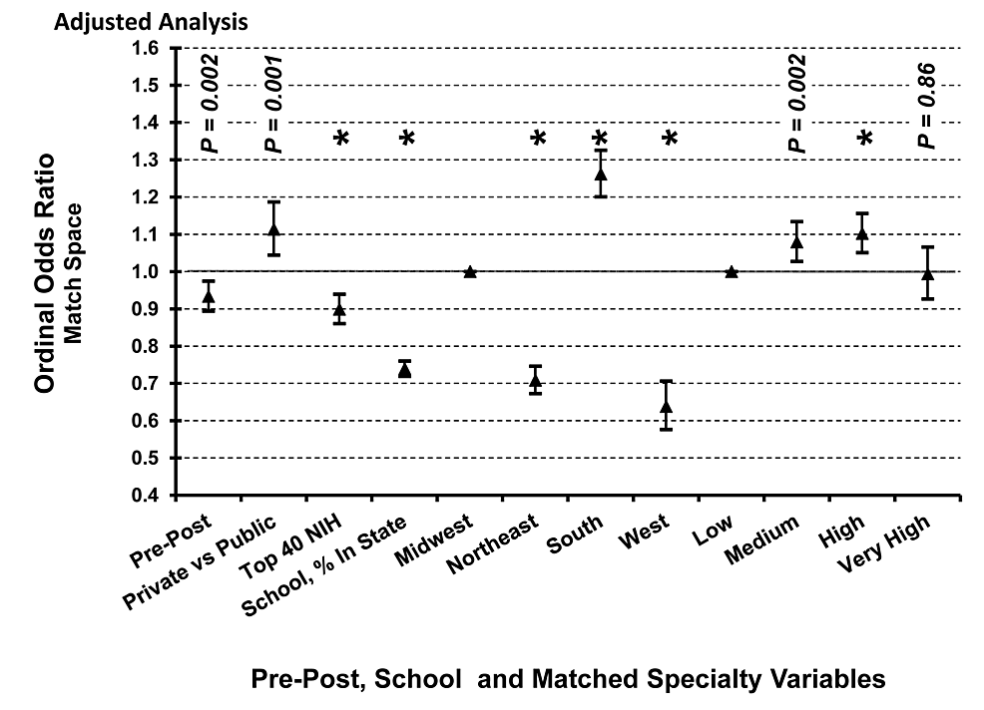
Relationship Between Match Space, School Characteristics, and Specialty Competitiveness, Pre- and Post-COVID-19. Graduating US medical school seniors’ match space. Values indicate odds ratios (triangles) and 95% confidence intervals adjusted by the other variables using ordinal logistic regression of match space (1 = home institution; 2 = home state; 3 = adjacent state; 4 = the same or adjacent US Census division (and non-adjacent state); 5 = skipped at least one US Census division). Asterisks indicate a *P* < 0.001. Variables include pre-post COVID-19 pandemic, private vs. public medical school designation, school from top 40 NIH funding, percentage of in-state matriculants (collapsed into quartiles), geographic location (vs. Midwest), and specialty competitiveness (vs. low competitiveness). Specialty competitiveness was determined based on predicted value using Factor Analysis: very high (plastic surgery, neurosurgery, dermatology, orthopedic surgery, otolaryngology), high (urology, radiation oncology, internal medicine, cardiothoracic surgery, general surgery), medium (diagnostic radiology, vascular surgery, obstetrics/gynecology, emergency medicine, anesthesiology), and low (pediatrics, neurology, psychiatry, pathology, family medicine, physical medicine, and rehabilitation). NIH = National Institute of Health

The match space after virtual interviews (post-COVID-19) was lower than during in-person interviews (pre-COVID-19) (OR 0.93, 95% Confidence Interval (CI), 0.89 to 0.97). Match space was also lower for graduates of top NIH institutions (OR 0.90, 95% CI, 0.86 to 0.94), schools with a higher percentage of in-state matriculants (OR 0.74, 95% CI, 0.72 to 0.76), schools from the Northeast or West as compared to the Midwest (OR 0.71, 95% CI, 0.67 to 0.75; OR 0.64, 95% CI, 0.58 to 0.71; respectively), see Figure 2. The match space was higher for students graduating from private institutions (OR 1.11, 95% CI, 1.04 to 1.19) and from the South as compared to the Midwest (OR 1.26, 95% CI, 1.20 to 1.33), see Figure 2.

As compared with less competitive specialties (referent), the match space was higher for students applying to medium competitive specialties (OR 1.08, 95% CI, 1.03 to 1.13) or high competitive specialties (OR 1.10, 95% CI, 1.05 to 1.16), but not for very high competitive specialties (OR 0.99, 95% CI, 0.93 to 1.07), see Figure 2.

## Discussion

In this analysis of 34,672 medical students, we explored geographic trends of residency placement for all specialties before and after the onset of the COVID-19 pandemic. Using the “match space” metric to quantify the geographic relationship between medical schools and residency programs, we found an overall decrease in match space during the COVID pandemic when compared to prior years; however, match space varied significantly depending on the characteristics of the medical school and specialty.

School characteristics

For the 2021 application cycle, graduates of private medical schools were more likely to match farther from their home institution than those from public schools, and graduates of schools with higher in-state percentages matched closer than those with lower percentages. This is consistent with pre-pandemic findings and with previous years and is reassuring for public schools, which often have a mission to develop physicians for their state [24,25].

We used NIH funding as a surrogate of school reputation when considering how reputation may impact match space. In the 2021 application cycle, graduates of the top 40 NIH-funded schools matched closer to their home institution, also consistent with pre-pandemic match trends. We suspect this is because top NIH-funded schools often have strong residency options that attract internal applicants.

Finally, we examined the US census region of each medical school to determine the match distance relative to other regions. Interestingly, applicants from the South had the highest match space, whereas the Northeast and West had lower match space relative to the Midwest. This finding suggests that applicants from the South match into programs significantly farther away from their home institution compared to applicants from other regions. This may reflect the density of training programs; however, further study is needed to determine why southern medical schools appear to have a lower retention rate of their graduates.

Specialty characteristics

Specialties were categorized by competitiveness from low to very high based on exploratory factor analysis including salary, US-senior fill rate, step 1 United States Medical Licensing Examination (USMLE) average, step 2 USMLE average, AOA percentage of matched applicants, and percentage of matched applicants from a top 40 NIH-funded school. In our analysis, we found medium and highly competitive specialties match farther compared to the least and most competitive specialties. We suspect that applicants applying to the most competitive specialties are more likely to match at their home program, which may mitigate some of the effects of fewer residency programs and positions that would otherwise increase match space.

Limitations

We recognize some limitations in our study. First, we included schools with publicly available match information, which could introduce selection bias and limit generalizability; however, we obtained a large sample size of individual matches from both public and private medical schools over four years. Second, match location depends on a mutual agreement between both the program and applicant, and thus we are unable to determine how much match distance is attributable to the student or residency program. This may be an area of further study by adding student and program rank lists in addition to match lists.

Implications

Other studies have investigated the geographic impact of the pandemic with mixed results but were either limited to a single specialty or dichotomized by in-state vs. out-of-state [12,26,27]. As programs and leaders in medical education grapple with decisions about away rotations and virtual or in-person interviews, they must consider safety, cost, and equity for all applicants against the backdrop of growing application inflation [28,29]. US graduates applied to an average of 73 programs in 2021 compared to 65 in 2019, and international medical graduates applied to an average of 147 programs compared to 137 in 2019 [30]. An additional, and perhaps less recognized, factor to consider is the geographic implication of these decisions on applicants. The piloting of secondary applications within certain specialties shows a growing recognition of the importance of match geography; however, additional research and understanding are needed.

## Conclusions

After the COVID-19 pandemic, students graduating from US allopathic schools matched closer to their home institution. Students attending public schools, schools with more in-state matriculants, and schools with higher research rankings also matched closer to their home institutions. Specialty competitiveness also impacted match distance; as compared with less competitive specialties, the match space was higher for students applying to medium competitive specialties (Diagnostic Radiology, Vascular Surgery, Obstetrics/Gynecology, Emergency Medicine, Anesthesiology) or high competitive specialties (Urology, Radiation Oncology, Internal Medicine, Cardiothoracic Surgery, General Surgery). We included more specialties than any other published study and more nuance in the measurement of match distance through the calculation of match space.
